# Systematic Coronary Risk Evaluation 2 (SCORE2), arterial stiffness, and subclinical coronary atherosclerosis in a population-based study

**DOI:** 10.1080/02813432.2025.2456948

**Published:** 2025-01-24

**Authors:** Susanna Strömberg, Andreas Stomby, Jan Engvall, Carl Johan Östgren

**Affiliations:** a Department of Health, Medicine and Caring Sciences, Linköping University, Linköping, Sweden; b Råslätts vårdcentral, Region Jönköping County, Jönköping, Sweden; c Centre of Medical Image Science and Visualization (CMIV), Linköping University, Linköping, Sweden

**Keywords:** Cardiovascular risk, coronary atherosclerosis, arterial stiffness, cardiovascular disease, subclinical vascular damage

## Abstract

**Aim:**

To investigate the association between Systematic Coronary Risk Evaluation 2 (SCORE2) and subclinical damage in two vascular beds: atherosclerosis in the coronary arteries and aortic arterial stiffness, in a large population-based cohort without cardiovascular disease or diabetes.

**Methods:**

*Design:* A cross-sectional study based on Swedish CArdio Pulmonary bioImaging Study (SCAPIS) data. *Study population:* A population-based cohort of 3087 participants aged 50–64.

**Outcome:**

Pulse Wave Velocity (PWV) was measured, and aortic arterial stiffness was defined as PWV≥ 10 m/s. Coronary artery calcium score (CACS) was determined by coronary computed tomography and clinically significant coronary calcification was defined as CACS > 100.

**Results:**

The prevalence of arterial stiffness was 6.6% in the low-moderate SCORE2 risk group, 31.0% in the high-risk group, and 53.3% in the very high-risk group. The prevalence of coronary calcification was 4.5%, 18.5% 23.0%, respectively. There was a modest overlap between arterial stiffness and coronary calcification in all SCORE2 risk groups. When comparing the high SCORE2 risk group with the low-moderate risk group, the Odds ratio (OR) was 6.4, 95% confidence interval (CI 5.1–8.0) for arterial stiffness and 4.8 (CI 3.7–6.3) for coronary calcification. When comparing the very high SCORE2 risk group to the low-moderate group, the OR was 16.2 (CI 11.3–23.1) for arterial stiffness and 6.4 (CI 4.2–9.7) for coronary calcification.

**Conclusion:**

Our study shows that high cardiovascular risk according to SCORE2 is associated with increased arterial stiffness and significant coronary calcification in a population without prevalent cardiovascular disease or diabetes. This knowledge can be useful in primary care, where SCORE2 is frequently used as a risk prediction tool. The modest overlap between arterial stiffness and coronary calcification suggests that CACS and PWV describe different types of vascular damage.

## KEY POINTS

What is known?

Arterial stiffness and coronary calcification are measures of subclinical vascular damage and predictors of poorer cardiovascular outcomes.

What is the knowledge gap?

There is limited data on the association between SCORE2 and subclinical macrovascular damage.

What this study shows?

This study shows an association between SCORE2 risk and arterial stiffness and subclinical coronary atherosclerosis. The higher the SCORE2 risk the higher the prevalence of both arterial stiffness and coronary calcification. These two measures overlap, albeit to a small extent.

## Background

Cardiovascular disease (CVD) is a major cause of mortality and disability worldwide [1,2]. Early detection of cardiovascular risk factors and subclinical damage to the vascular organs can reduce a person’s cardiovascular risk through preventive measures [3–5]. Established cardiovascular risk factors serve as essential components in predictive models that can be used to estimate the probability of a cardiovascular event within a given period. Among these models, the Systematic Coronary Risk Evaluation (SCORE) algorithm is a widely used tool in Europe to estimate the 10-year risk of cardiovascular mortality in people aged 40–69 years without diabetes or previous cardiovascular disease. In 2021, an updated algorithm, SCORE2, was introduced that uses contemporary data to estimate the 10-year risk of fatal and non-fatal cardiovascular events [6]. This updated algorithm is based on age, sex, smoking status, blood pressure, non-high density lipoprotein (non-HDL), and European region of residence for predictions.

Subclinical coronary atherosclerosis is common in the general population [7]. It can be visualised by non-contrast cardiac computed tomography, which shows coronary artery calcification quantified by the coronary artery calcification score (CACS) [8]. Another sign of subclinical vascular damage is increased arterial stiffness. This is characterized by reduced compliance of the blood vessel wall in response to blood pressure [9]. Carotid femoral pulse wave velocity (PWV) is widely considered the gold standard for assessing arterial stiffness [10]. Elevated PWV signifies vascular damage within the aorta and is closely associated with increased cardiovascular risk [11].

Increased arterial stiffness and CACS are associated with each other in the general population [12]. Both measures are independent predictors of CVD, extending their predictive value beyond established risk assessment tools such as SCORE and the Framingham Risk Score and are therefore relevant measures of subclinical vascular damage [13–15].

However, the relationship between arterial stiffness and subclinical coronary atherosclerosis compared to estimated cardiovascular risk according to the updated SCORE2 algorithm has not yet been investigated.

Thus, the aim of this study was to investigate the association between cardiovascular risk according to SCORE2 and subclinical vascular damage in two vascular beds: the coronary arteries and the aorta, in a large population-based cohort without CVD or diabetes.

## Methods

### Participants

**Figure 1.**
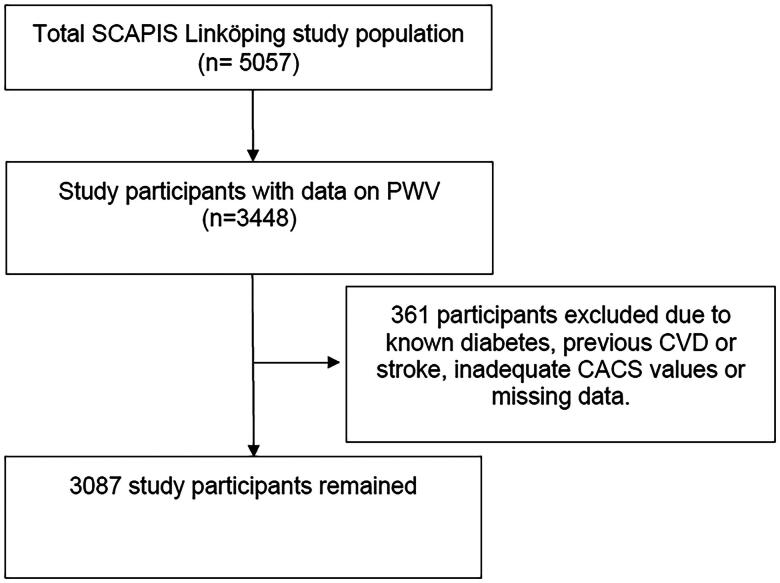
Flowchart of participants.

The study was conducted as part of the Swedish CardioPulmonary BioImage Study (SCAPIS), a prospective study comprising a population-based cohort of 30,000 participants at six sites in Sweden. Detailed information about the study has been described previously [7,16]. In brief, participants in this study were randomly selected from the population of Linköping municipality aged 50–64 years, and the examinations were performed at Linköping University Hospital. The only exclusion criterion was the inability to understand Swedish and the participation rate was 58%. Of the 5057 study participants, 3087 remained for analyses due to different reasons clarified in Figure 1. The data was collected between 2015 and 2018.

Ethical approval for SCAPIS was granted by the Regional Ethical Review Board (Dnr 2018/478-31) and the study complied with the principles outlined in the Declaration of Helsinki. Written informed consent was obtained from all participants.

### Measurement of cardiovascular risk factors and covariates

This assessment included a questionnaire of self-reported health status, medical history, medication use, lifestyle choices, occupation, and social determinants. Venous blood samples were taken after an overnight fast to collect data on plasma glucose, HbA1c, lipids, and creatinine levels. Standard anthropometric measurements were also performed. Blood pressure was measured twice in each arm using an automated device (Omron M10-IT, Omron Healthcare Co, Kyoto, Japan). M10-IT, Omron Healthcare Co, Kyoto, Japan).

### Pulse wave velocity

Pulse wave velocity (PWV) was not part of the core study protocol, but an additional substudy at Linköping University. PWV was measured using the Sphygmocor Xcel applanation tonometer (Atcor Medical, Australia). Blood pressure cuffs were applied to the right thigh (10–20 cm below the groin) and the left upper arm. At the same time, a carotid tonometer was used to record the blood pressure waveforms at the carotid artery. The distance between the carotid and femoral sites was measured to facilitate the calculation of PWV [12]. A PWV cut-off value of >10 m/s was used to define arterial stiffness, following established standards [10].

### Coronary artery calcification

Coronary artery calcification was assessed using non-contrast-enhanced images from a coronary computed tomography scanner (Siemens, Somatom Definition Flash Siemens Healthineers, Erlangen, Germany), as previously described [7,16]. Coronary calcification was quantified in terms of Agatston units according to international standards and the proprietary software ‘Syngo Calcium Scoring’ (Siemens Healthineers) was used [8]. Clinically relevant coronary artery calcification was defined as CACS > 100 according to the guidelines for the categorisation of study participants with moderate to severe coronary plaque [17].

### Statistical methods

Continuous data were presented as mean (SD) and categorical data as number of individuals and proportion. CACS was negatively skewed and therefore analysed as ordinal data. A scatterplot was created to visualise the relationship between SCORE2 risk and PWV. Chi-square tests were used for comparisons with categorical variables, while one-way ANOVA tests and post hoc Bonferroni tests were used for comparisons with mean values.

Univariate logistic regression models were used to calculate odds ratios for PWV >10 and CACS> 100 as a function of SCORE2 risk group. Linear models with SCORE2 as a continuous variable were also used. Multivariate models were adjusted for BMI and educational level. Venn diagrams were used to visualise PWV >10 m/s and CACS >100 for low to moderate SCORE2 risk, high SCORE2 risk, and very high SCORE2 risk and to show how these overlap [18].

A *p*-value of <0.05 was considered statistically significant. SPSS version 29 was used for the statistical calculations.

## Results

Participants with a history of diabetes or CVD or with missing data on the variables included in SCORE2 were excluded, resulting in a study population of 3087 participants (Figure 1). The characteristics of the study participants are shown in Table 1. The mean PWV was 8.82 m/s, and arterial stiffness (PWV > 10 m/s) was present in 17.5% of the total population. Approximately 37% had a CACS > 0, with 10% exceeding a CACS > 100. The prevalence of both arterial stiffness and coronary calcification (CACS > 100) increased concomitantly with the SCORE2 risk categories (Table 1).

**Table 1.** Characteristics of participants according to SCORE2 risk group.

	Total population	Low to moderate risk <5%	High risk 5– < 10%	Very high risk ≥10%	*p* Value for difference between SCORE2 risk groups
Sociodemographic					
Sample size	3087	1851 (60.0)	1071 (34.7)	165 (5.3)	N/A
Female	1594 (51.6)	1298 (70.1)	279 (26.1)	17 (10.3)	N/A
Age years	57.3 (4.4)	55.6 (3.9)	59.5 (3.9)	61.5 (3.2)	N/A
Current smoker	286 (9.3)	65 (3.5)	155 (14.5)	66 (40.0)	N/A
Highest level of education University	1348 (43.7)	892 (48.2)	398 (37.2)	58 (35.2)	*p* = 0.001^*,#^
Clinical chemistry					
Cholesterol mmol/L	5.6 (1.0)	5.5 (1.0)	5.7 (1.1)	5.9 (1.1)	N/A
HDL mmol/L	1.7 (0.5)	1.8 (0.5)	1.5 (0.4)	1.3 (0.3)	N/A
LDL mmol/L	3.4 (0.9)	3.2 (0.9)	3.6 (1.0)	3.8 (1.0)	N/A
hCRP mg/L	1.8 (3.6)	1.6 (3.0)	2.0 (3.3)	2.7 (6.3)	*p* = 0.001*^#^
HbA1c mmol/mol	35 (4)	35 (3)	36 (4)	38 (11)	*p* = 0.001*^#†^
eGFR mL/min/1.73 m [2]	81 (12)	82 (12)	82 (12)	79 (12)	*p* = 0.008^#^ *p* = 0.026^†^
Medical history					
Currant treatment for hyperlipidaemia	128 (4.1)	54 (2.9)	64 (6.0)	10 (6.1)	*p* = 0.001*
Currant treatment hypertension	464 (15.0)	196(10.6)	225 (21.0)	43 (26.1)	*p* = 0.001*^#^
Heredity for cardiovascular disease *n* (%)	219 (7.1)	143 (7.7)	64 (6.0)	12 (7.3)	*p* = 0.206
Anthropometry and biological measures					
BMI, kg/m [2]	26.4 (3.9)	25.7 (3.9)	27.3 (3.7)	28.0 (3.8)	*p =* 0.001*^#^
Systolic blood pressure, mm Hg	132 (17)	126 (15)	140 (16)	153(19)	N/A
Diastolic blood pressure, mm HG	83 (10)	80 (9)	86(10)	92 (10)	N/A
Pulse	61 (9)	61 (9)	62 (9)	63 (12)	*p =* 0.009*^#^
PWV m/s	8.82 (1.31)	8.36 (1.09)	9.41 (1.28)	10.13 (1.39)	*p =* 0.001*^#†^
PWV > 10 m/s	550 (17.5)	122 (6.6)	332 (31.0)	88 (53.3)	*p =* 0.001*^#†^
CACS >0	1147 (37.2)	483 (26.1)	565 (52.8)	99 (60)	*p =* 0.001*^#^
CACS > 100	319 (10.3)	83 (4.5)	198 (18.5)	38 (23.0)	*p =* 0.001*^#^
CACS >400	83 (2.7)	13 (0.7)	59 (5.5)	11 (6.7)	*p =* 0.001*^#^

Abbreviations: HDL: high-density lipoprotein cholesterol; LDL: low-density lipoprotein cholesterol; hCRP: High-sensitivity C-reactive protein; HbA1c: haemoglobin A1c; eGFR: estimated glomerular filtrations rate; BMI: body mass index; PWV: pulse wave velocity; CACS: coronary artery calcium score. Continuous data are presented with mean and standard deviation. Categorical data are presented with number of individuals and per cent. Normally distributed values are compared with an ANOVA test and categorical data with a chi-square test. Bonferroni was used for adjustments for multiple comparisons.

*Low-moderate SCORE2 risk group vs high SCORE risk group.

^#^Low-moderate SCOER2 risk group vs very high SCORE risk group.

^†^High SCOER2 risk group vs very high SCORE risk group.

**Figure 2.**
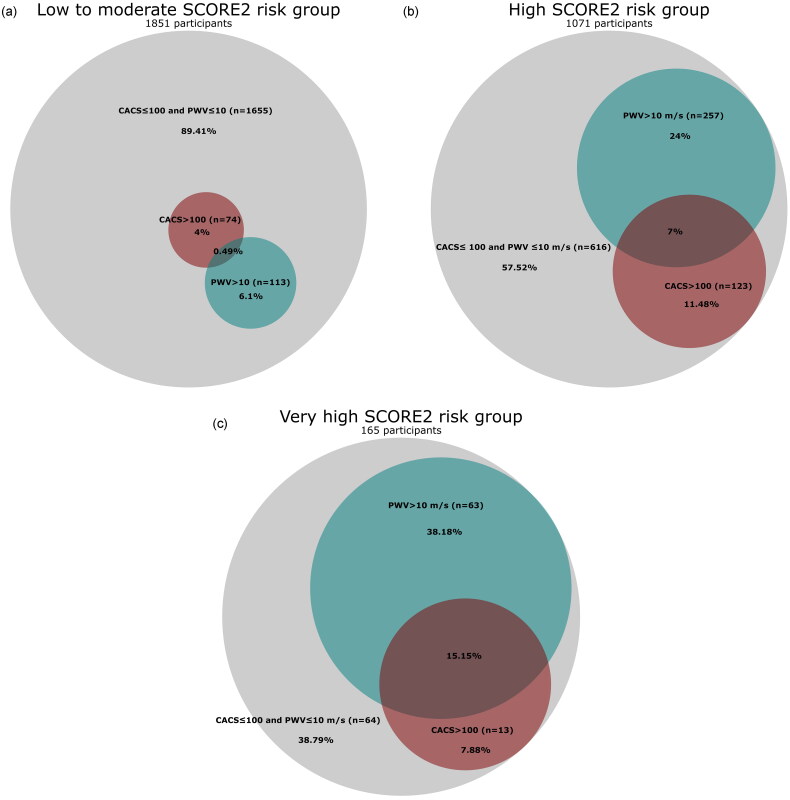
Venn diagram of the distribution of coronary artery calcium score (CACS >100) in red, pulse wave velocity (PWV >10 m/s) in green in (a) low to moderate SCORE2 risk group, (b) high SCORE2 risk group and (c) very high SCORE2 risk group.

Of the total population, 26% had either arterial stiffness, coronary calcification, or both, indicating subclinical vascular damage. When the population was stratified by SCORE2 risk, the prevalence of subclinical vascular damage was 11%, 42%, and 61% in the groups with low-moderate, high, and very high SCORE2 risk respectively, with a significant difference between groups (*p* = 0.001) (Figure 2). 26% of individuals with subclinical vascular damage had a low-moderate risk, 60% had a high risk and only 15% had a very high risk. In all risk groups, the prevalence of arterial stiffness was higher than that of coronary calcification, but this difference was only statistically significant in the high-risk group (*p* = 0.02).

**Figure 3.**
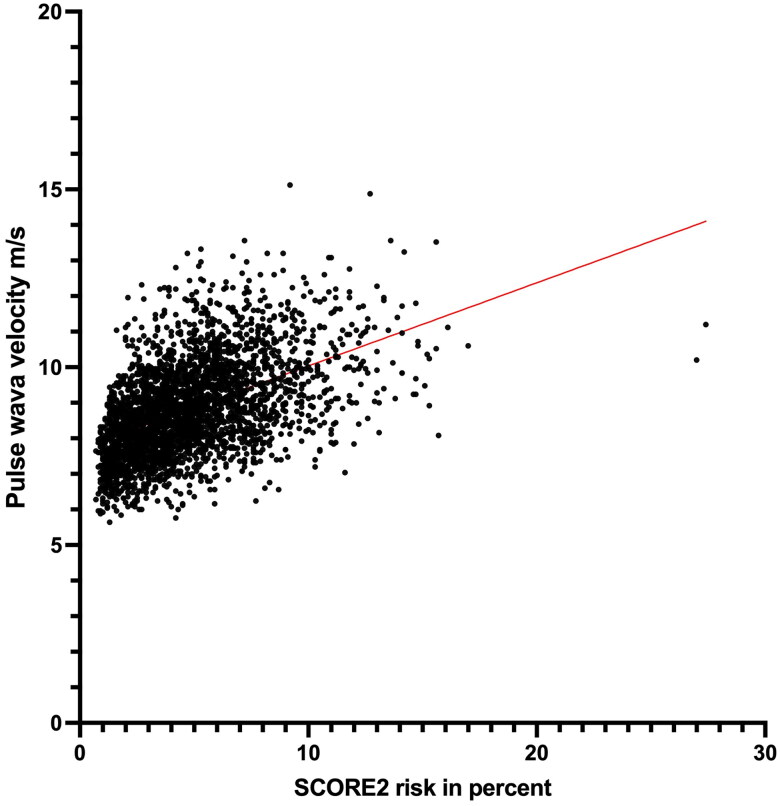
Scatterplot of SCORE2 and PWV.

The SCORE2 risk correlated with the PWV (*R* = 0.495, R2 = 0.245, *p* = 0.001 Figure 3). In a linear regression model, PWV increased by 0.2 m/s (95% CI 0.2–0.2) for each percentage point increase in SCORE2 risk (*p* = 0.001) (data is not shown in table). This relationship remained statistically significant after adjustment for BMI and education level. In a logistic regression model comparing the high SCORE2 risk group with the low to moderate SCORE2 risk group, the odds ratio for arterial stiffness was 6.4 (CI 5.1–8.0) and for coronary calcification 4.8 (CI 3.7–6.3). When comparing the very high risk group with the low to moderate risk group, the odds ratio was 16.2 (CI 11.3–23.1) for arterial stiffness and 6.4 (CI 4.2–9.7) for coronary calcification (Table 2). In a logistic regression model with SCORE2 as a continuous variable, the odds ratio for arterial stiffness (PWV > 10 m/s) increased by 1.4 (CI 1.4–1.5) for each percentage point increase in SCORE2 risk. The corresponding odds ratio for coronary calcification (CACS > 100) was 1.3 (CI 1.2–1.3) (Supplementary Table 1).

**Table 2.** Logistic regression of comparing low risk SCORE2 to group moderate and high risk SCORE2 risk to PWV > 10 and CACS> 100.

	PWV> 10 m/s		CACS> 100	
	Odds Ratio (95% CI) Crude	Odds Ratio (95% CI) Adjusted for BMI and education level	Odds Ratio (95% CI)	Odds Ratio (95% CI) Adjusted for BMI and education level
Low to moderate risk group				
High risk group	6.4 (5.1–8.0)	5.9 (4.7–7.5)	4.8 (3.7–6.3)	4.6 (3.5–6.0)
Very high-risk group	16.2 (11.3–23.1)	14.7 (10.2–21.1)	6.4 (4.2–9.7)	5.9 (3.9–6.1)

For the logistic regression models, a sensitivity analysis was performed excluding individuals with a medical history of hyperlipidaemia and hypertension, leaving the results virtually unchanged (Supplementary Table 2).

## Discussion

In this population-based study, we found a clear association between increased cardiovascular risk according to SCORE2 and the prevalence of arterial stiffness and subclinical coronary atherosclerosis in a general population without diabetes or established CVD. As expected, there was an overlap between arterial stiffness and atherosclerosis, and this overlap increased with increasing SCORE2 risk. Notably, this overlap remained relatively modest, suggesting that CACS and PWV describe different types of vascular damage. SCORE2 is not intended to be used for risk estimation in patients with diabetes and prevalent cardiovascular disease. Accordingly, as we excluded people with these diseases in this study, only a small proportion, 5.3%, was categorised as very high risk.

To our knowledge, this is the largest epidemiological study examining the SCORE2 in relation to arterial stiffness and subclinical coronary atherosclerosis in a large random sample of the general population without diabetes or CVD. A recent SCAPIS study presented the prevalence of coronary atherosclerosis in different SCORE2 risk groups and CACS > 0 in relation to carotid plaque and microcirculation [19]. In contrast, the focus of our study is macrovascular disease by including arterial stiffness. The results are consistent with previous population studies that have demonstrated an association between CACS and various cardiovascular risk assessments such as the SCORE and the Framingham Risk Score [14,15]. SCORE estimates the risk of cardiovascular mortality within 10 years, while SCORE2 estimates the 10-year risk of fatal and non-fatal cardiovascular events. The risk group categorisations are different for SCORE and SCORE2. SCORE has four categories: low, moderate, high and very high. SCORE2 has three categories: low to moderate, high and very high. In a previous study within the SCAPIS cohort on SCORE and subclinical atherosclerosis identified a prevalence of CACS ≥ 100 of 2% in the low-risk group, 14% in the moderate-risk group and 32% in the high/very high risk group [14]. In our study we observed a prevalence of CACS > 100 of 4.5% in the low to moderate risk group and 18% in the high risk group and 23% in the very high risk group. SCORE2 categorises more people into the high and very high risk groups compared to SCORE and the prevalence of objective signs of vascular damage is lower in these risk groups than in the SCORE stratification.

A previous SCAPIS study investigating arterial stiffness and subclinical atherosclerosis in different glycaemic categories reported the presence of subclinical vascular damage in the early stages of dysglycaemia, characterised by a modest overlap between arterial stiffness and coronary calcification in all glycaemic categories [20]. This observation is consistent with our results, where we also found limited overlap between arterial stiffness and subclinical atherosclerosis, but in our study the extent of overlap increased with the increase in SCORE2 risk groups. A lower cut-off value such as CACS > 0 would result in a higher prevalence of subclinical atherosclerosis and may even result in a greater extent of overlap [21,22]. We opted for CACS > 100 as this has a higher specificity for subclinical atherosclerosis and is thus more clinically relevant.

The difference between subclinical vascular damage in the aorta and the coronary arteries is noteworthy. These vascular beds are characterised by different morphological features that could explain the differences. Arterial stiffness primarily reflects the ageing of the aorta, a large artery with morphological changes occurring predominantly in the tunica media and characterised by increased collagen content and decreased elastin content. In contrast, coronary calcification occurs in medium-sized arteries, with the atherosclerotic process occurring mainly in the tunica intima. These processes are linked, as increased arterial stiffness leads to increased luminal pressure and shear stress, which in turn leads to endothelial dysfunction. This in turn accelerates the development of atheromas and excessive collagen deposition in the tunica intima of the arterial wall, which eventually leads to atherosclerosis [11]. Interestingly, PWV does not increase in the early stages of atherosclerosis, which are characterised by increased thickness of the intima or non-calcified atheromas. However, in the presence of atherosclerotic plaques, PWV increases [9]. Furthermore, PWV measures arterial compliance, whereas CACS assesses morphological changes. It is noteworthy that the parameters used in the SCORE2 algorithm, such as smoking status, lipid levels, systolic blood pressure and sex, together influence several parts of the cardiovascular system [23].

## Strengths and limitations

The greatest strength of this study is the large population-based sample. However, the strengths of this study are balanced by some limitations. There is a risk of selection bias, as low socioeconomic status was associated with lower participation rates in the SCAPIS study [24]. Furthermore, while CACS provides valuable insights into atherosclerotic burden, it does not provide information on plaque stability, which could be a limitation. In addition, CACS only quantifies calcified plaque and does not account for non-calcified plaque. However, previous studies within the SCAPIS cohort have shown that non-calcified plaques are rare in this population [7]. Due to the cross-sectional design, no conclusions on causality can be drawn from this study.

## Clinical implications

According to the European guidelines, CACS can be used to guide treatment decisions in addition to traditional risk factors in individuals at intermediate or borderline risk [25]. However, it is important to consider not only the economic resources but also the time required by healthcare professionals to implement these guidelines [26]. A time needed to treat calculation provides a broader perspective on the resources needed for a new implementation. Prioritizing resources becomes important. However, we are not advocating the inclusion of CACS and PWV in clinical practice. This study investigated the relationship between SCORE2 and subclinical vascular injury using these measures. Knowledge of the prevalence of arterial stiffness and subclinical atherosclerosis at different estimated SCORE2 risk levels could influence physician interventions and further diagnostic investigations at the individual level. In the very high SCORE2 risk group, which represents 5% of the apparently healthy population, two-thirds already had subclinical vascular damage indicating the need of preventive measures. This knowledge can serve as a valuable tool for physicians and patients at very high risk of CVD, potentially motivating lifestyle changes and improving medication adherence.

In the low to moderate SCORE2 risk group, only one in ten people had subclinical vascular damage, confirming a lower CVD risk in this group. The challenge lies in the high-risk group, which accounts for 35% of the total population and 61% of those with subclinical vascular damage. Therefore, a risk assessment tool to better identify people with subclinical vascular damage within the high-risk group could be beneficial. However, it is important to consider the actual risk in per centage and not only the risk group category, as there is a wide range of subclinical vascular damage within the high-risk group.

## Conclusion

This study shows a clear association between cardiovascular risk according to SCORE2 and arterial stiffness and subclinical coronary atherosclerosis in a general population without CVD. The prevalence of vascular damage was low in the low to moderate SCORE2 risk group and high in the very high SCORE2 risk group. This knowledge could be useful in general practice for the assessment and prevention of cardiovascular risk. Although there was an overlap between arterial stiffness and coronary calcification, this overlap remains relatively small in all SCORE2 risk groups.

## Supplementary Material

Supplemental Material
