# Supplementary material for: Systematic Coronary Risk Evaluation 2 (SCORE2), arterial stiffness, and subclinical coronary atherosclerosis in a population-based study
Source: Scand J Prim Health Care. 2025 Jan 24;43(2):455–62. doi: 10.1080/02813432.2025.2456948 (PMC12090275; doi:10.1080/02813432.2025.2456948)
Supplement: Supplemental Material [file IPRI_A_2456948_SM5943.docx]

## Supplementary

Table 1

Logistic regression comparing SCORE2 risk to PWV>10 and CACS> 100

|  | PWV> 10m/S |  | CACS> 100 |  |
| --- | --- | --- | --- | --- |
|  | Odds Ratio (95% CI) Crude | Odds Ratio (95% CI) Adjusted BMI and education level | Odds Ratio (95% CI) | Odds Ratio (95% CI) Adjusted for BMI and education level |
| SCORE2 | 1.42(1.37-1.47) | 1.40 (1.35-1.45) | 1.28 (1.23-1.33) | 1.20(1.23-1.33) |

Table 2: Logistic regression SCORE2 and PWV> 10 and CACS >100 the study population excluding participants with current hypertension and hyperlipidaemia treatment (n=2567)

|  | PWV> 10m/S |  | CACS> 100 |  |
| --- | --- | --- | --- | --- |
|  | Crude | Adjusted for BMI and education level | Crude | Adjusted for BMI and education level |
| Low-moderate risk group |  |  |  |  |
| High risk group | 6.7 (5.3-8.9) | 6.5 (4.9-8.5) | 5.0 (3.3-6.3) | 4.5 (3.3-6.3) |
| Very high-risk group | 19.8 (13.0-30.2) | 18.4 (12.0-28.1) | 5.9(3.5-10.0) | 5.8 (5.4-10.0) |
| SCORE2 as a continuous variable | 1.4 (1.4-1.5) | 1.4 (1.4-1.5) | 1.2(1.2-1.3) | 1.2 (1.2-1.3) |
